# Predictors of insecticide-treated nets utilization among children under five years in refugee settlements in Uganda: analysis of the 2018–2019 Uganda Malaria Indicator Survey

**DOI:** 10.1186/s12936-025-05262-4

**Published:** 2025-01-21

**Authors:** Henry Musoke Semakula, Frank Mugagga

**Affiliations:** Department of Geography, Geo-Informatics and Climatic Sciences, Makerere University, P.O Box 7062, Kampala, Uganda

**Keywords:** Children under five, Insecticide-treated nets, Malaria, Malaria indicator survey, Refugees, Settlements, Uganda

## Abstract

**Background:**

Despite significant distribution of insecticide-treated net (ITNs) by the Government of Uganda to refugees, malaria is major cause of mortality and morbidity among children under five years in refugee settlements. This highlights the persistent challenges and complexities surrounding malaria control and prevention efforts in these settings. Studies that focus on the determinants of ITN utilization among children under five years in refugee settlements in Uganda are not available. Using the 2018–2019 Uganda’s Malaria Indicator Survey (UMIS) data, analysis of the individual and household factors associated with utilization of ITN among children under five in refugee settlements of Uganda was conducted.

**Methods:**

This study focused on 589 children under five staying in refugee settlements located in Uganda. The extracted variables from the UMIS included social-economic factors associated with ITN utilization. Descriptive analysis was performed to generate summarized statistics, while inferential statistics by way of bivariate analysis were performed to assess the association between the outcome and the independent variables using the chi-square test, and multivariable logistic regression modelling to assess the magnitude of the associations after controlling for other covariates. All analyses considered the survey sampling design and sampling weights, and are conducted in Stata version 18.

**Results:**

The odds of children sleeping under ITN were higher if their mothers had secondary and higher education (8.1 times) as well as primary education (1.5 times). The odds of children sleeping under ITN reduced by 50% if their mothers were pregnant. Interestingly, the odds of children sleeping under ITN were 70% lower if their mothers knew that ‘not sleeping in nets’ caused malaria. Mothers who were exposed to malaria messages had lower odds of their children sleeping under ITNs.

**Conclusions:**

The results highlight areas of intervention that can increase ITN use in refugee settlements of Uganda. Improving access to education for mothers, providing targeted health education on the importance of ITN, dispelling misconceptions about malaria transmission, facilitating the proper installation of ITNs among others, can all contribute to increased ITN utilization among children under five.

## Background

Malaria remains a significant global health and development challenge, disproportionately affecting vulnerable populations, particularly in sub-Saharan Africa [1]. Among the vulnerable populations, children under the age of five are the most susceptible to severe malaria and its associated morbidity and mortality due to their underdeveloped immune systems [2]. In refugee settings of Uganda, the burden of malaria among children is exacerbated by factors such as overcrowded living conditions, inadequate healthcare access, and limited resources for preventive measures [3]. The most common malaria vectors in Uganda with preference to temporary water bodies and permanent water bodies, are *Anopheles gambiae* Complex (*Anopheles gambiae *sensu stricto (*s.s.*) and *Anopheles arabiensis*). *Anopheles funestus*, with *Anopheles gambiae* being the dominant species in most locations [4]. A recent study conducted across nine refugee settlements in Uganda where these misquotes are found, indicates that malaria prevalence among children under five years is 32.8% [3] and this underscores the critical need for targeted interventions such as insecticide-treated nets (ITNs).

The use of ITNs is one of the most effective ways to prevent malaria transmission and has proven to show significant reduction in malaria morbidity and mortality across a range of transmission settings in Africa [5–7]. ITNs have the ability to kill or repel mosquitoes which feed and rest indoors as well as preventing night mosquito bites. By 2022, a total of 254 million ITNs were distributed in all malaria endemic countries with about 235 million (93%) distributed in sub-Saharan Africa (SSA) [1]. Half of the ITNs distributed to SSA were received by six countries which include the Democratic Republic of the Congo (33.6 million), Nigeria (28.4 million), Ethiopia (21.4 million), Sudan (18.9 million), Uganda (13.8 million) and Mali (12.5 million) [1]. The Government of Uganda adopted the policy of mass distribution of ITNs as one of the significant interventions for malaria prevention. Three mass campaigns across Uganda including refugee settlements, have already been implemented in 2013–2014, 2017–2018, and 2020–2021 [8]. On arrival, the refugees are given free ITNs by United Nations High Commissioner for Refugee (UNHCR) in collaboration with Uganda’s Malaria Control Division of the Ministry of Health. The typical strategy allocates one ITN per two sleeping spaces within a household. For instance, a household with five members might receive two or three nets depending on sleeping arrangements. Since 2023, over, 1.1 million ITNs have successfully been distributed to refugees [9].

Despite global efforts to scale up ITN distribution in such contexts, utilization rates often fall short of expectations, influenced by various social, behavioral, and environmental predictors. Studies have shown that the key determinants of ITN utilization in SSA include hot weather, absence of visible mosquitoes, poor attitude to use ITNs, lack of ownership of ITN, negligence among households, suffocation caused by ITNs, and unpleasant odour associated with ITNs among others [10]. In Uganda, the factors influencing the use of ITNs include: access to ITNs, age of household head, sex of household head, number of sleeping rooms, wealth, malaria prevalence, mother’s level of education, mother’s knowledge of malaria transmission, residence, and region [11–13].

While previous studies have explored ITN usage in general populations of sub-Saharan Africa [10], there is limited evidence specifically addressing refugee contexts, where unique challenges and opportunities may influence utilization patterns. Uganda, a malaria-endemic country hosts one of the largest refugee populations in the world making it an ideal setting for this investigation. This study aimed to fill this knowledge gap by examining the factors associated with ITN use among young children in refugee settlements. This study has contributions to the growing body of literature on malaria and has some policy implications. This is the first study to concentrate on the utilization of ITN in refugee settlements of Uganda based on nationally representative datasets. Studying the determinants of ITN utilization in these unique communities, can lead to a better understanding of the actions that can increase ITN use in these settings.

## Methods

### Conceptual framework

**Fig. 1:**
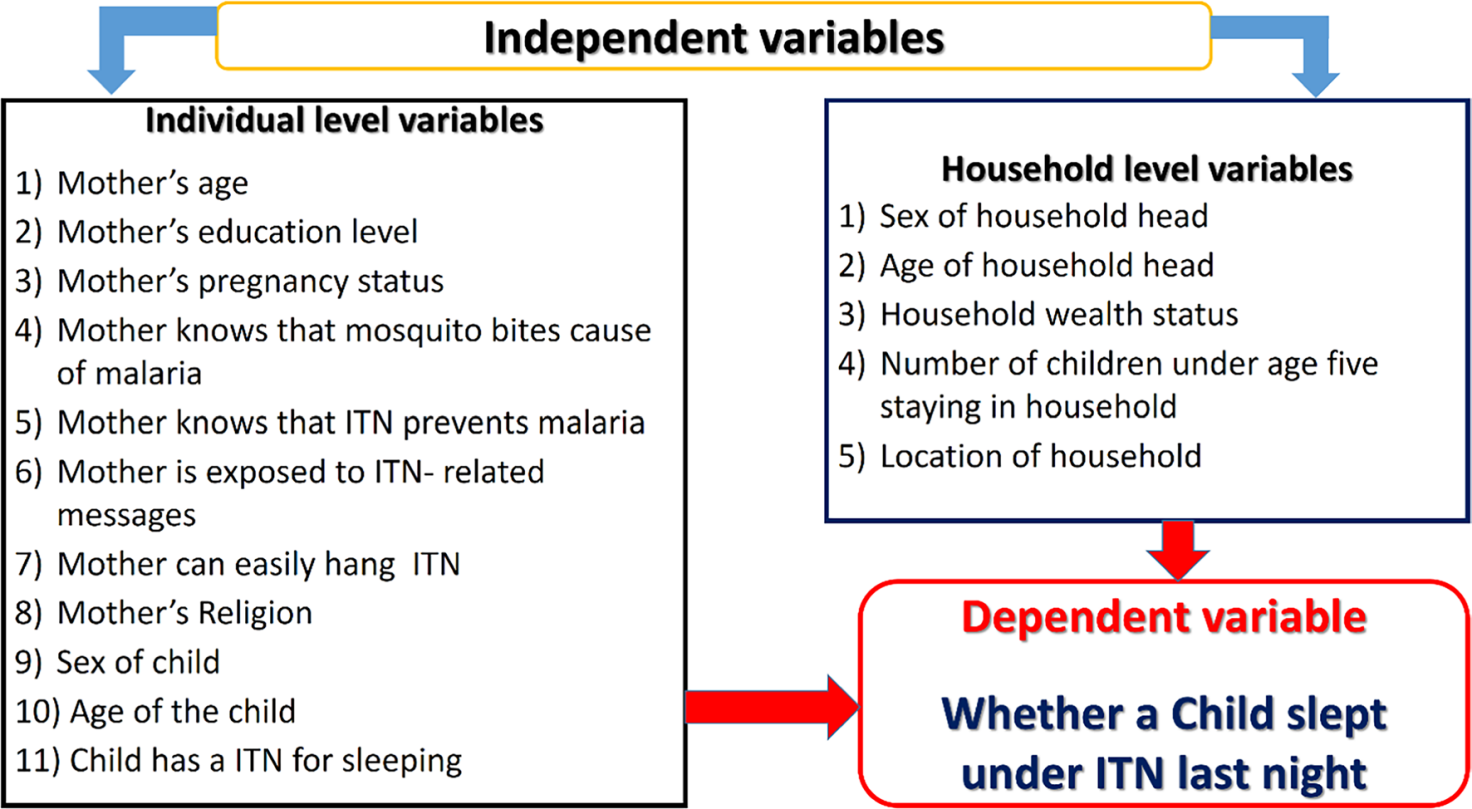
Conceptual framework

A conceptual framework (Fig. 1) was developed to provide a visual representation of the key variables and their relationships within this study. In this study, the outcome of interest was whether a child under five years of age living in refugee settlements of Uganda slept under ITN the previous night. The explanatory variables deemed relevant to construct the conceptual framework for this study were chosen from a review paper which focused on identifying factors associated with ownership and utilization of ITNs among children under five years in SSA [10]. The identified variables were categorized into two levels; that is individual and household as shown in Fig. 1. The conceptual framework structure was based on literature review, expert knowledge, and previous malaria modelling experience in refugee settlements [14].

### Study location

**Fig. 2:**
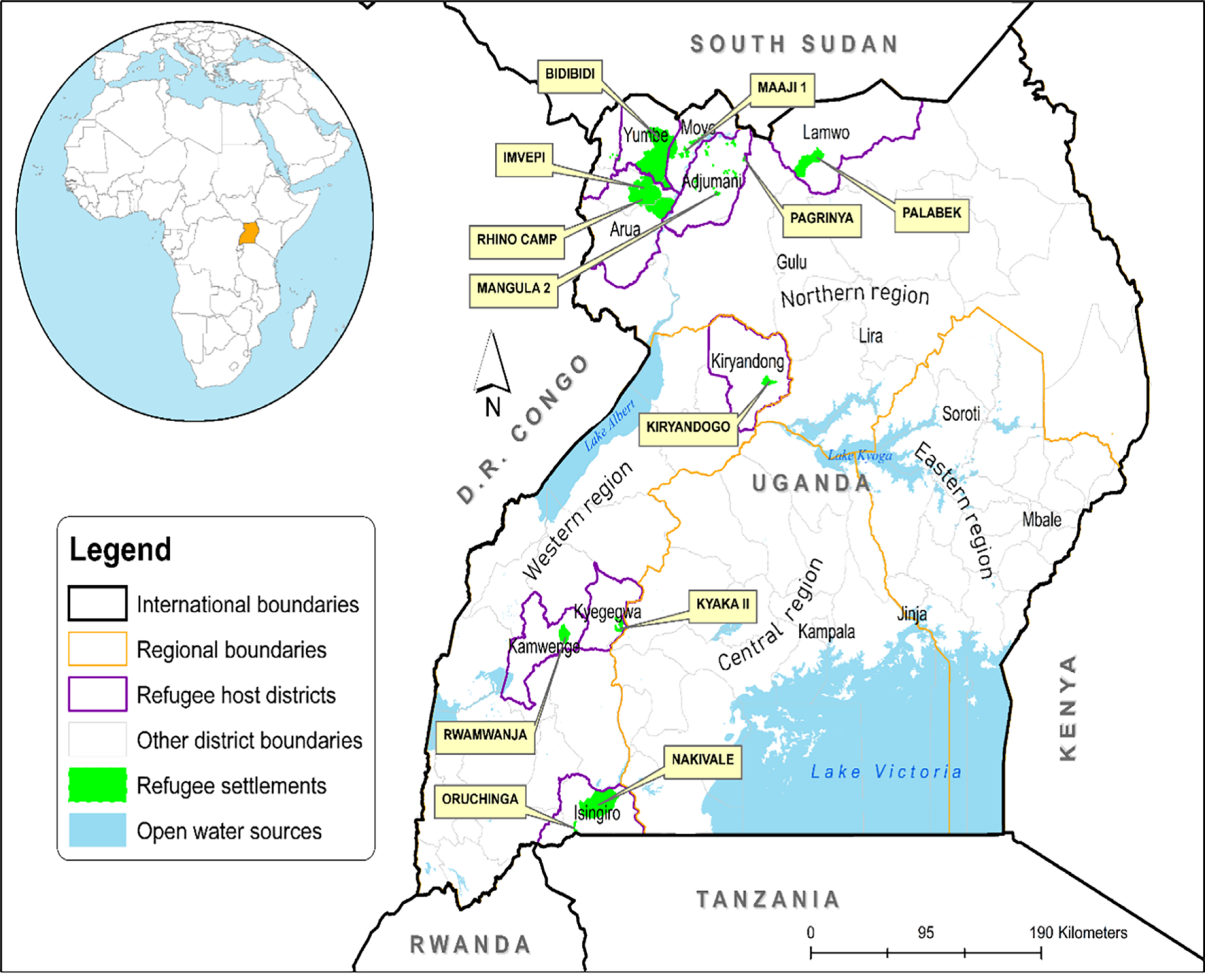
Location of refugee settlements in Uganda[3]

The study is a secondary analysis of the 2018–2019 Malaria Indicator Survey of Uganda focusing on 12 refugee settlements in Uganda (Fig. 2). The refugees in Uganda come from various neighbouring countries in East Africa and the Great Lakes region, primarily due to political instability, persecution, ethnic tensions, human rights abuses, and calamities. By 2023, South Sudan made up the largest refugee population in Uganda (57%), followed by the Democratic Republic of Congo (D.R. Congo) (31%), while other refugees constituting 12% came from Somalia, Rwanda, Burundi, and Sudan [15]. Most of the refugees (92%) live in settlement camps, while 8% live in Kampala, the capital city of Uganda.

### Data source

The 2018–2019 MIS is the third malaria survey conducted in Uganda. The MIS is among the DHS Program cross-sectional surveys, also a household based survey designed to collect data focused on internationally accepted malaria indicator*s****.*** This survey has specific malaria questions and malaria parasitaemia testing. The survey was conducted between December 2018 and February 2019 at the peak of malaria transmission season shortly after the rainy season. The MIS collected data on the availability and use of ITNs in households, and several other malaria indicators in refugee settlements of Uganda. The sampling frame was based on Uganda’s Population and Housing Census frame for 2014 and this survey employed a two-stage stratified sampling procedure. In the first stage, stratification of the sampling frame by geographic area (urban, rural and refugee) was conducted. The stratification was based on the enumeration areas of Uganda’s population census of 2014. Within the first stage, two activities were done: selection of clusters from each strata and listing of households in the selected clusters. In the second stage, selection of households to be interviewed was accomplished. In this study, the unit of analysis was children of women born in the last 5 years (0–59 months). This age group is particularly vulnerable to a range of health, nutrition, and developmental challenges and it is a standard focus age group for comparability across studies and regions. The DHS dataset used in this study was the Kids Recode (KR file) and it contained all the variables captured in the conceptual framework (Fig. 1). This dataset was downloaded from The DHS Program website (https://dhsprogram.com/). A total of 7124 children were included in this survey. Since the focus of this study was children in refugee settlements, other children in non-refugee camps where excluded. After weighting and excluding children with missing responses (67), the sample size for this study was 589 children.

### Dependent variable

The dependent variable for this study was whether a child under 5 slept under ITN last night before the survey. Respondents who indicated that their children under 5 slept under treated mosquito net the night before the survey were considered as using ITN and coded as 1, whereas those children under 5 who did not sleep under net were considered as not using ITN and were coded as 0.

### Independent variables

As indicated in the conceptual framework (Fig. 1), two level variables (individual and household) associated with utilization of ITNs were identified from empirical evidence [10] and were fully captured within the KR file of the MIS dataset.

### Individual level factors

Three variables related to the child of interest were considered and these included; possession of ITN categorized as yes and no; age of child categorized as 0–11 months, 12–24 months, and 25–60 months and sex of the child categorized as male and female. Eight variables related to the primary caregivers (mothers) were considered and these included; age of mothers, education level, pregnancy status, mother's religion, not sleeping in net causes malaria, exposure to malaria messages, mosquito bites cause malaria, and the ease to hang a bet net by mothers. Age of mothers was categorized as less than 24, 25–34, 35–49. Mothers’ education was measured as highest level of educational attainment and categorized into no education, primary, secondary and higher. Pregnancy status was classified as pregnant and not pregnant. Mother’s religion was categorized as Christians (i.e. Catholics, Anglican, Orthodox, Pentecostal, Jewish, Baptist) and Muslims. Four variables: not sleeping in net causes malaria, exposure to malaria messages, mosquito bites cause malaria, and the ease to hang a bet net by mothers had binary responses and were categorized as yes and no.

### Household level factors

Five variables were considered at the household level and these included; sex of household head categorized as male and female; age of household head classified as less than 30 years, 31–39 years, above 40 years; region of residence categorized as northern region (i.e. included all households located in Adjumani, Arua, Moyo, Yumbe and Lamwo districts) and Southern region (i.e. included all households in Kamwenge, Isingiro, Kiryandongo, and Kyegegwa districts); number children staying in household categorized as 1 child, 2–3 children, 3 above and household wealth index. The household wealth index was derived from aggregation of household’s asset possession. Assets such as type of floor/roofing, radio, television, car, toilet and source of water were computed into wealth index using principal component analysis and categorized into poorest, poorer, middle, richer and richest. These were re-categorized into poor middle and rich. Multicollinearity was checked on all the variable using the pwcorr tab command of Stata software and the variables which had correlation coefficients of 0.5 and above, were considered as highly correlated and were excluded from the analysis. The variables of TV and radio ownership were correlated with household wealth and were excluded from the analysis.

### Statistical analysis

In this paper, three types of analyses were performed. These included descriptive analysis of the data resulting into summarized statistics, bivariate analysis to assess the association between the outcome and the independent variables using the chi-square test, and multivariable logistic regression modelling to assess the magnitude of the associations after including controls. The inclusion criteria variables in multivariable logistic regression was set at *P* < 0.50). This higher significance level (*P* < 0.50) for variable selection and inclusion in the multivariable logistic regression analysis was considered to void excluding known important variables [16] relevant to the outcome variable of this study with a small sample size of 589. Three multivariable logistic regression models were performed to cater for (1) individual level factors, (2) household level factors and (3) a combination of both the individual and household level factors. Odds ratios were estimated with corresponding 95% confidence intervals. To account for disproportionate sampling and nonresponse, DHS weights provided as six-digit integers in the KR file dataset were normalized (i.e. divided by 1,000,000) and used to provide weights to the data [17]. To adjust for the effect of the complex survey design, the DHS variables of primary sampling unit, strata and sampling weights were used in the analysis. All statistical analyses were conducted in Stata version 18 (StataCorp, College Station, TX, USA).

## Results

### Descriptive statistics of the respondents

Table 1 presents ITN use among refugee children under 5 Ugandan by socio-demographic characteristics at the individual, household and region level as captured by the conceptual framework in Fig. 1. A total of 589 children under 5 living in refugee settlements were included in this study. For the outcome variable, 68.8% of the children in refugee settlements slept under an ITN on the night before the survey. A higher proportion of children sleeping under ITN was recorded in households located in the northern districts (72.3). Generally, 86.3% of the children had an ITN for sleeping. Children aged less than 11 months were 24.3%, those between 12 and 24 months were 37.2% and those above 25 months were 38.5%. In this study, 52.6% of the children were females while 52.6% of the mothers to the children were aged between 25 and 34 years. Additionally, 47.2% of the mothers had no formal education. Mothers who did not know that ‘not sleeping in nets’ caused malaria were 93.9%. Besides, 75.7% of the mothers were exposed to malaria messages. Although majority of the mothers were exposed to malaria messages, while 78.9% didn’t know that mosquito bites cause malaria infection. Six in 10 of households were headed by women. Most of the households had two children (70.1%). Moreover, 45.2% of the household heads were less than 30 years of age while others aged between 31–39 and above 40 years, were 33.1 and 21.7%, respectively. A majority of the households (93.7%) were categorized as poor.

**Table 1:** Characteristics of the study population

Variables	Location of refugee settlements		
	All n (%)	Northern n (%)	Southern n (%)
Child's characteristics Age in months			
0–11	143 (24.3)	97 (24.0)	46 (24.9)
12–24	219 (37.2)	153 (37.9)	66 (35.7)
25–59	227 (38.5)	154(38.1)	73 (39.5)
Sex of child			
Male	279 (47.4)	191 (47.3)	88 (47.6)
Female	310 (52.6)	213 (52.7)	97(52.4)
Child has ITN for sleeping			
No	77 (13.7)	47(11.6)	26 (41.1)
Yes	486 (86.3)	357(88.4)	159 (85.9)
Child slept under the net last night			
No	184 (31.2)	112 (27.7)	72 (38.9)
Yes	405 (68.8)	292 (72.3)	113 (61.1)
Number children staying in household			
One child	98 (16.6)	67 (16.6)	31 (16.8)
Two children	413 (70.1)	269 (66.6)	144 (77.8)
Three children and above	78 (13.2)	68 (16.8)	10 (5.4)
Mother's characteristics Age of mothers (years)			
Less than 24	153 (26.0)	123 (30.5)	30 (16.20
25–34	310 (52.6)	206 (51.0)	104 (56.2)
35 and above	126 (21.4)	75 (18.6)	51 (27.6)
Mother's educational level			
No education	278 (47.2)	164 (40.6)	114 (61.6)
Primary	263 (44.7)	208 (51.5)	55 (29.7)
Secondary and higher	48 (8.2)	32 (7.9)	16 (8.7)
Mother’s pregnancy status			
Not pregnant	544 (92.4)	383 (94.8)	161 (87.0)
Pregnant	45 (7.6)	21 (5.2)	24 (13.0)
Not sleeping in net causes malaria			
No	553 (93.9)	392 (97.0)	161 (87.0)
Yes	36 (6.1)	12 (3.0)	24 (13.0)
Exposure to malaria messages			
No	446 (75.7)	278 (68.8)	168 (90.8)
Yes	143 (24.3)	126 (31.2)	17 (9.2)
Mosquito bites cause malaria			
No	465 (78.9)	321 (79.5)	144 (77.8)
Yes	124 (21.1)	83 (20.5)	41 (22.2)
Mother's religion			
Muslim	56 (9.5)	24 (5.9)	32 (17.3)
Christians	533 (90.5)	380 (94.1)	153 (82.7)
Ease of hanging a bet net			
No	13 (2.2)	8 (2.0)	5 (2.7)
Yes	576 (97.8)	396 (98.0)	180 (97.3)
Household characteristics Sex of household head			
Male	235 (39.9)	133 (32.9)	102 (55.4)
Female	354 (60.1)	271 (67.1)	83 (44.9)
Age of household head (Years)			
Less than 30	266 (45.2)	196 (48.5)	70 (37.8)
31–39	195 (33.1)	120 (29.7)	75 (40.5)
40 +	128 (21.7)	88 (21.8)	40 (21.6)
Household wealth status			
Poor	527 (93.7)	391 (96.8)	165 (89.2)
Rich	36 (6.3)	13 (3.2)	20 (10.8)
Languages spoken/understood			
English	64 (10.9)	55 (13.6)	9 (4.9)
Luo	28 (4.8)	22 (5.5)	6 (3.2)
Runyankole	36 (6.1)	–	36 (19.6)
Lugbara	27 (4.6)	27 (6.7)	–
Others	434 (73.7)	300 (74.30	134 (72.4)
Total	589 (100.0)	404 (68.6)	185 (31.4)

## Bivariate analysis

The test of association between the dependent variable (ITN use) and the independent variables was performed using the Chi-square test and the results are shown in Table 2. From Table 2, only five independent variables (Child’s age, mother’s education, pregnant status, knowledge on causes of malaria and ownership of television) were significantly associated with the dependent variable (child slept under ITN last night). Utilization of ITN was most common among children less than 11 months (76.7%) and children between 12 and 24 years. Besides, the use of ITN among children less than five years was higher among women with secondary and higher levels of education compared to those who did not have any formal education (62.3%). The use of ITN among children was higher among women who were not pregnant (69.5%) compared to those who were pregnant (44.4%). Additionally, the use of ITN of children under 5 was also highest among mothers who didn’t know that ‘not sleeping in nets’ caused malaria (68.5% compared to those who knew (45.9%). Finally, the level ITN utilization was highest among households which had no television (67.4%) compared to those who had (33.1%).

**Table 2:** Association between child slept under net last night and child's, mother's and household's level characteristics

Variables	%	CI	*P* value
Individual level factors a) *Child's characteristics*			
Age in months			< 0.001
0–11	76.7	[63.7,86.1]	
12–24	69.6	[60.0,77.7]	
25–59	58.1	[46.0,69.2]	
Sex of the child			0.488
Male	65.1	[54.3,74.4]	
Female	68.1	[55.9,78.2]	
Child has ITN for sleeping			0.548
No	22.8	[17.4,29.4]	
Yes	77.2	[70.6,82.6]	
b) *Mother’s characteristics*			
Age of mothers (Years)			0.437
Less than 24	65.7	[45.7,81.3]	
25–34	69.4	[56.4,79.8]	
35 and above	61.9	[40.2,79.7]	
Mother's educational level			0.004
No education	62.3	[50.9,72.5]	
Primary	67.8	[57.5,76.6]	
Secondary and higher	90.8	[67.7,97.9]	
Mothers’ pregnancy status			0.003
Not pregnant	69.5	[60.6,77.0]	
Pregnant	44.4	[26.4,64.0]	
Not sleeping in net causes malaria			0.003
No	68.5	[58.8,76.9]	
Yes	45.9	[31.0,61.6]	
Exposure to malaria messages			0.048
No	67.2	[57.6,75.6]	
Yes	63.7	[45.8,78.5]	
Mosquito bites cause malaria			0.427
No	65.3	[54.0,75.1]	
Yes	71.4	[55.6,83.3]	
Mother's religion			0.623
Muslim	71.1	[50.5,85.5]	
Christians	66.2	[55.1,75.8]	
Ease of hanging a bet net			0.028
No	56.7	[26.1,83.0]	
Yes	66.8	[56.3,75.9]	
Household characteristics			
Sex of household head			0.464
Male	65.6	[53.9,75.6]	
Female	68.1	[55.8,78.4]	
Age of household head (Years)			0.490
Less than 30	68.9	[57.5,78.4]	
31–39	68	[54.8,78.8]	
40+	60.2	[41.6,76.4]	
No	66.3	[57.6,74.0]	
Yes	67.6	[45.0,84.2]	
Household wealth			0.945
Poor	66.6	[56.1,75.6]	
Rich	67.2	[44.0,84.3]	
Region of residence			0.126
Northern districts	73.1	[61.7,82.0]	
Southern districts	59.4	[44.1,73.1]	
Number children staying in household			0.837
One child	67	[48.4,81.5]	
Two children	67.4	[57.4,76.1]	
Three children and above	61.1	[32.2,83.9]	

### Determinant of ITN utilization among children less than five years

A multivariable logistic regression analysis was performed to determine the magnitude of the associations between the independent variables and the outcome variables and results are captured in Table 3.

**Table 3:** Adjusted logistic regression for the predictor of ITN utilization among children in refugee settlements

Variables	Dependent variable: child slept under net last night					
	Combined individual and household characteristics		Individual characteristics		Household characteristics	
	AOR	95% CI	AOR	95% CI	AOR	95% CI
Child age in months (Ref. less than 11)						
12–24	0.6	0.4 –1.0	0.7	0.5 – 1.2	–	–
25–59	0.4***	0.3 –0.5	0.4***	0.3 – 0.6	–	–
Sex of the child (Ref. Male)						
Female	1.5	1.0 –2.2	1.3	0.9 – 2.0	–	–
Age of mothers (Years) (Ref. Less than 24)						
25–34	1.5	0.5 – 4.3	1.4	0.5 – 4.3	–	–
35–49	1.9	0.5 –7.1	1.2	0.4 – 3.6	–	–
Mother's educational level (Ref. None)						
Primary	1.5*	1.0 –2.3	1.5*	1.0 –2.1	–	–
Secondary and higher	8.5***	2.7—26.8	8.1***	2.9—22.8	–	–
Mother’s pregnancy status (Not pregnant)						
Pregnant	0.5	0.2 – 1.2	0.5*	0.2 –1.0	–	–
Not sleeping in net causes malaria (Ref. No)						
Yes	0.3*	0.1 – 0.8	0.3*	0.1–0.9	–	–
Exposure to malaria messages (Ref. No)						
Yes	0.5*	0.3 – 1.0	0.7	0.5 –1.1	–	–
Mosquito bites cause malaria (Ref. No						
Yes	1.1	0.5 – 2.6	1.1	0.5 – 2.5	–	–
Ease of hanging a bet net (Ref. No)						
Yes	5.9**	1.6 –21.5	3.7*	1.2 –11.3	–	–
Sex of household head (Ref. Male)						
Female	0.5	0.3 – 1.0	–	–	0.6*	0.3 – 1.0
Age of household head (Years) (Ref. < 30)						
31–39	0.9	0.5 –1.7	–	–	1	0.5 – 2.1
40 +	0.5*	0.2 – 1.0	–	–	0.6	0.3 – 1.1
Region of residence (Ref. Southern districts)						
Northern districts	2.4*	1.2 –4.7	–	–	2.4*	1.2 –4.7

*AOR*  adjusted odds ratio

***P < 0.001, **P < 0.01, *P < 0.05

In Table 3, children aged between 25 and 59 months had 60% lower odds of sleeping under ITN compared to the reference category (less than 11 months). The odds of children sleeping under ITN were higher if their mothers had secondary and higher education (8.5 times) as well as primary education (1.5 times) compared to children whose mothers had no education. The odds of children sleeping under ITN reduced by 50% if their mothers were pregnant, compared to children whose mothers were not pregnant. Surprisingly, the odds of children sleeping under ITN were 70% lower if their mothers knew that ‘not sleeping in nets’ caused malaria compared to those who didn’t know. Higher odds of children sleeping under ITN were observed if their mothers were able to hang the net with ease (5.9 times). The odds of children sleeping under ITN reduced by 40% among female headed households compared to male headed households. Besides, the odds of children sleeping under ITN were 2.4 times higher in households located in refugee settlements of the northern districts of the compared to households located in the refugee settlements in the southern districts.

## Discussion

This study aimed to provide an overview of the determinants of ITN utilization among children under 5 in refugee settlements of Uganda using the 2018–2019 Malaria Indicator Survey datasets which is nationally representative. Overall, 68.8% of children under five years slept under an ITN in households on the night before the survey. This percentage is moderate given the high levels of ITN distribution in Uganda, through various universal coverage campaigns in 2013–2014, 2017–2018, and 2020–2021 [18]. This percentage finding is however lower than that of a study conducted in Soroti District, North Eastern Uganda, where it was observed that 89.4% of children slept under the bed net the previous night to the survey, a figure that is far much higher than the national estimates [13]. However, disparities in usage remain, with challenges in consistent use among certain demographics and geographic regions. For instance, women and children in rural and less-advantaged areas often face barriers to ITN access and utilization [19]. The study established significant factors associated with the use of ITNs for malaria control among children under 5 years in refugee settlements in Uganda.

### Age

Age of the child was key in influencing ITN utilization. The results show that older children (25–59 months) had a 60% lower odds of utilizing ITN and this is in-line with findings by another study done to evaluate bed net use in Soroti District, North Eastern Uganda [13]. Possible explanations to this results could be that older children in refugee settlements are viewed as being immune to malaria infections and as such, don’t require ITN for sleeping at night. Besides, in cases of fewer ITNs, young children could be given priority compared to older one. This finding is also consistent with other studies done in sub-Saharan Africa in regard to factors influencing the use of bed nets by children under 5 years [10].

### Mother’s education

The results further indicate that children whose mothers had some formal education had higher odds of sleeping under ITNs compared to children whose mothers had no education. This result implies that is educated mothers can make better decisions about health outcomes of children compared to uneducated ones. For example, there are more likely to understand the risks of malaria, protective benefits of ITNs, and their proper usage. Similar significant associations between formal education and sleeping under ITNs among children under five years have also been observed in other studies [10, 12, 20]. Thus, investing in education initiatives for mothers in these populations can potentially improve ITN coverage and contribute to the prevention of malaria and other vector-borne diseases. Additionally, health promotion campaigns should be tailored to target less-educated mothers in these refugee settlements, using visual aids and simplified language to overcome literacy barriers.

### Mothers’ pregnancy status

The results of this study further indicated that children whose mothers were pregnant had lower odds of sleeping in ITN compared to children whose mothers were not pregnant. This result imply that refugee families might incorrectly assume that children are at lower risk of malaria than pregnant women, leading to reduced focus on protecting children with ITNs. Similar observations have been made in other studies done in sub-Saharan Africa were pregnant women always prioritize other aspects of prenatal care (e.g., clinic visits, supplements) over ITN use among their children under five years, especially if they believe that other malaria prevention strategies (e.g., indoor spraying, anti-malarial medications) are sufficient [10]. Moreover, when pregnant women go for antenatal care (ANC), a lot of emphasis is put on both maternal and fetal health rather than their children under five years [21]. Thus, health workers in ANC health facilities should also emphasis the importance of ITN for children of pregnant mothers.

### Malaria awareness among mothers

The odds of children sleeping under ITNs were 70% lower when their mothers were aware that ‘not sleeping under ITN’ could cause malaria, compared to mothers who were unaware of this fact. There are possible explanations to this result. First, this results may suggest a problem of mothers’ understanding of the content of health messaging and this calls for a health literacy assessment around malaria prevention messages. Second, even if mothers in refugee settlements understood the importance of ITNs, factors such as cost, unavailability, or inconvenience of setting up nets might prevent their use. Besides, maternal knowledge and beliefs about malaria transmission and prevention may play a significant role in shaping mothers’ behaviors related to ITN use among their children. Addressing misconceptions and promoting accurate knowledge about malaria transmission and the effectiveness of ITNs could be crucial strategies for improving ITN utilization and reducing the burden of malaria among children under five years in refugee settlements of Uganda.

### Ease of hanging ITNs

The findings suggest that the ease with which mothers were able to hang ITNs contributed to increased usage of these nets among their children. This highlights the importance of considering practical factors such as accessibility and convenience when implementing malaria prevention interventions, particularly in refugee settings. It should be noted that ITNs that are easy to hang or set up may encourage daily use, as they require less time and effort to install. This is especially important in busy households, where mothers may prioritize tasks that save time or reduce complexity. Moreover, if mothers find it difficult or time-consuming to set up ITNs, they may be less likely to use them consistently. Simplifying the process can remove one of the key barriers to ITN utilization, especially in households with lower literacy or limited resources. Thus, providing support and resources to facilitate the proper installation and maintenance of ITNs can enhance their effectiveness as a tool for preventing malaria transmission among children under five years.

### Mother’s exposure to malaria messages

The findings suggest that mothers who were exposed to malaria messages had lower odds of their children sleeping under ITNs. This result may indicate a potential discrepancy between the messaging content or delivery and its effectiveness in promoting ITN use among mothers in refugee settings. It should be noted that refugees who come to Uganda speak Arabic or French yet these malaria messages are given in English. Thus, there is a possibility that these refugees do not understand the English-based malaria messages. Moreover, the malaria messages may not address the barriers some refugee households face in acquiring or using ITNs. Nevertheless, further exploration is needed to understand why exposure to malaria messages did not translate into increased ITN usage and to identify potential barriers or factors influencing this relationship. Adjustments to messaging strategies or targeted interventions may be necessary to improve the uptake of ITNs among children in these populations.

### Location of refugee settlement

In addition, the odds of children sleeping under ITNs were 2.4 times higher in households located in refugee settlements of the northern districts compared to households located in refugee settlements in the southern districts. This significant difference underscores the importance of considering regional factors and geographical location when implementing malaria prevention interventions within refugee populations. There various possible explanations for this result. First, there is a high level of influx of refugees in the northern regions and this has made humanitarian organizations and local health authorities to prioritized ITN distribution. Second, the northern districts are more malaria-endemic than the southern districts, thus this could have driven more intense efforts to promote ITN usage, especially among vulnerable populations like children under five. Third, the communities in the northern refugee settlements may have greater awareness or more favourable attitudes toward ITN use. This could be the result of previous health education campaigns, peer influence, or higher levels of understanding about the importance of ITNs in malaria prevention. Understanding the specific challenges and barriers to ITN utilization in different areas can inform targeted strategies to improve coverage and promote consistent use of ITNs among children living in refugee settings across Uganda.

### Strengths and limitations of the study

This is the first study to provide an overview of ITN utilization among children under 5 in refugee settlements of Uganda using the MIS data which is nationally representative. DHS uses a complex sampling methodology, often based on multi-stage cluster sampling, which helps ensure that data are representative at the national, regional, and local levels. The study uses an adequate sample size, which renders the findings generalizable to the refugee children in Uganda. Despite these strengths, this study was not devoid of limitations.

First, the cross-sectional nature of the study design did not allow a causal-effect relationship to be established with certainty among the identified predictors; moreover, malaria incidence follows a seasonal pattern which the UMIS did not account for. Second, the MIS survey captured self-reported data and thus susceptible to various forms of selection biases, including coverage bias, non-response bias, respondent preference bias and recall bias particularly if pregnant women were less likely to remember or report using ITNs consistently among their children under five years of age. Third, the data set used lacked the qualitative insights, and as a result, mothers’ lived experienced as regards ITN utilization among their children were not captured. Fourth, certain variables (e.g., wealth proxies like TV/radio ownership were not fully able to capture the socio-economic disparities in refugee settings. Despite these limitations, the study was adequately powered to detect several important determinants of ITN utilization among children under five years.

## Conclusion

The study has indicated that individual and household level factors influence ITN use among children under 5 in refugee settlements of Uganda. Formal education among mothers, not being pregnant, the ease of hanging ITNs*,* location of households in northern districts of Uganda and a child being younger than 25 months increased prospects of ITN use among children under 5 in refugee settlements. While mother’s exposure to malaria messages, malaria awareness among mothers and being pregnant reduced greatly the prospects of ITN use among children under 5 in refugee settlements. These findings underscore the importance of considering these factors in the distribution and promotion of ITNs within refugee settlements. Tailored interventions that address specific barriers to ITN use, such as providing targeted health education, ensuring equitable access to education, and adapting distribution strategies to suit local contexts, are essential for improving ITN utilization among children in refugee settings. Future studies should focus on the determinants of ITN utilization for all refugees above 5 years since older children and adults are among the vulnerable populations.

## Data Availability

No datasets were generated or analysed during the current study.

## Abbreviations

DHS: Demographic and Health Surveys
ITNs: Insecticide-treated bed-nets
SSA: Sub-Saharan Africa
UMIS: Uganda Malaria Indicator Survey
WHO: World Health Organization
